# Nanotechnology: A Promising Approach for Delivery of Neuroprotective Drugs

**DOI:** 10.3389/fnins.2020.00494

**Published:** 2020-06-09

**Authors:** Saba Naqvi, Archna Panghal, S. J. S. Flora

**Affiliations:** Department of Pharmacology & Toxicology, National Institute of Pharmaceutical Education and Research, Raebareli, India

**Keywords:** nanotechnology, nanoformulations, targeted delivery, blood-brain barrier, neurological disorders, neuroprotection

## Abstract

Central nervous system (CNS) disorders especially neurodegenerative disorders are the major challenge for public health and demand the great attention of researchers to protect people against them. In past few decades, different treatment strategies have been adopted, but their therapeutic efficacy are not enough and have only shown partial mitigation of symptoms. Blood-brain barrier (BBB) and blood-cerebrospinal fluid barrier (BSCFB) guard the CNS from harmful substances and pose as the major challenges in delivering drugs into CNS for treatment of CNS complications such as Alzheimer’s disease (AD), Parkinson’s disease (PD), Huntington’s disease (HD), stroke, epilepsy, brain tumors, multiple sclerosis (MS), and encephalitis, etc. Nanotechnology has come out as an exciting and promising new platform of treating neurological disorders and has shown great potential to overcome problems related to the conventional treatment approaches. Molecules can be nanoengineered to carry out multiple specific functions such as to cross the BBB, target specific cell or signaling pathway, respond to endogenous stimuli, and act as a vehicle for gene delivery, support nerve regeneration and cell survival. In present review, the role of nanocarrier systems such as liposomes, micelles, solid lipid nanoparticles (SLNPs), dendrimers, and nanoemulsions for delivery of various neurotherapeutic agents has been discussed, besides this, their mechanism of action, and nanoformulation of different neuroprotective agents like curcumin, edaravone, nerve growth factors in CNS disorders like Alzheimer’s, Parkinsonism, epilepsy, stroke, and brain tumors has been reviewed.

## Introduction

**FIGURE 1:**
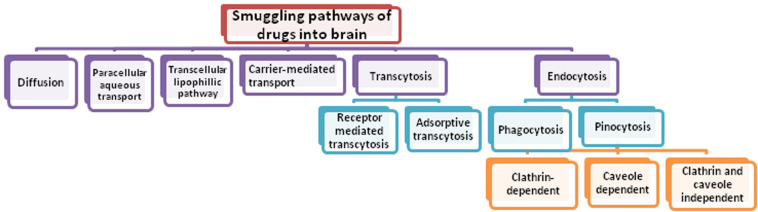
Schematic presentation of various transportation pathways across BBB.

Central nervous system (CNS) disease and disorders are posing great challenge for mankind and is the fastest growing area appealing for the attention of researchers. Today, neurological disorders are among the highest reasons for causing death and disability in the world. The contribution of CNS disorders to the global burden of disease is increasing and globally, in 2016, neurological disorders were the leading cause of disability-adjusted life-years (DALYs) [276 million (95% UI 247–308)] and second leading cause of deaths [9⋅0 million (8⋅8–9⋅4)] (Feigin et al., 2019). To date, the diagnosis and treatment of the neurological disorders such as Alzheimer’s disease (AD), Parkinson’s disease (PD), Huntington’s disease (HD), head trauma, brain tumor, and epilepsy are still a challenging task (Barchet and Amiji, 2009). A wide spectrum of potential drugs has been investigated to treat several neurological disorders but their therapeutic success is still limited due to range of challenges. One of the most common challenge is the difficulty in delivering agents such as drugs, nucleic acids, proteins, imaging agents and other macromolecules to the CNS across the peripheral barriers, namely blood-brain barrier (BBB) and blood-cerebrospinal fluid barrier (BCSFB), particularly the BBB (Pathan et al., 2009; Wong et al., 2012). Figure 1 showing different smuggling pathways of drug through which it may enter into the brain.

The current neurotherapeutics are associated with two major limitations i.e., before entering into brain cells they are pose to limited entry via BBB as insufficient entry in brain as well as of limited access to immune cells to brain (Misra et al., 2003; Nagpal et al., 2013). Most of the drugs used in neural diseases are lipophilic in nature having molecular weight greater 400–500 Da and not able to cross the BBB in pharmacologically significant quantity (Fischer et al., 1998) hence do not pass in initial screening whereas small lipophilic molecules (alcohol and steroid hormones) penetrate via transcellular mechanism. However, the presence of different transporters on endothelial cells which are highly selective in nature also limits the drugs entry. Also the presence of Tight junctions (TJ) in endothelial membranes creates high transendothelial electrical resistance (TEER) (>1000 W cm^2^) in cerebral microcapillaries (Gloor et al., 2001). The endothelial cells of BBB restrict the passage or entry of endogenic/foreign materials. ATP-binding cassette (ABC) transporters present over the endothelial cells and restrict the therapeutic drug entry also along with neurotoxic agents/metabolites/hormones/mediators. These transporters are membrane proteins require ATP to transport materials through cell membranes. Multiple drug resistance protein 1 (MDR1) or ATP-binding cassette sub-family B member 1 (ABCB1), which is well known as permeability glycoprotein (P-gp), multiple resistance protein 4 (MRP4) or ATP-binding cassette sub-family C member 4 (ABCC4), and breast cancer resistance protein (BCRP) or ATP-binding cassette sub-family G member2 (ABCG2) are members of this family (Alyautdin et al., 2014). The BBB also maintains the ionic homeostasis at synapses, where it regulates the entry of potassium ions (k^+^), calcium (Ca^2+^), sodium (Na^+^) and maintains minimum optic concentrations.

The BBB is made up of monolayer of polarized endothelial cells connected by complex tight junctions, and its functionality is controlled by cells such as astrocytes, neurons, and pericytes. The complexity of BBB, the existence of high levels of efflux transport proteins including P-glycoproteins (P-gp) and Multidrug Resistant Protein-1 (MRP-1), and the expression of metabolic enzymes limits the entry of drug inside the brain (Tajes et al., 2014).

**FIGURE 2:**
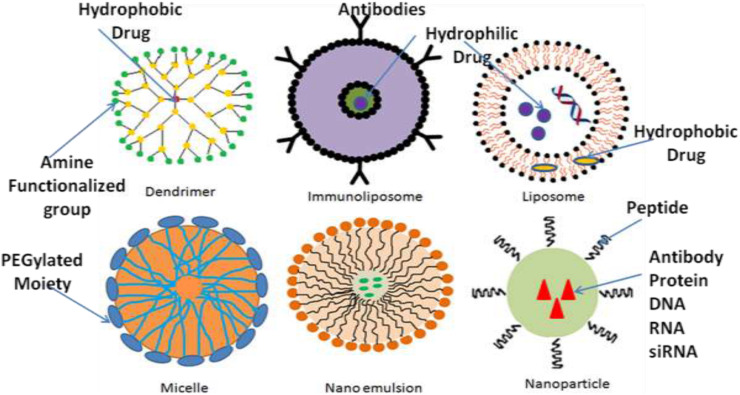
Nanotechnology-based various CNS delivery systems.

Recently, efforts such as alterations in the permeability efficiency of the BBB, carrier and receptor-mediated drug delivery, have been adopted to overcome the obstacles faced during successful treatment, even though the impact of most of these strategies has been found not much effective. Lipidization of a molecule with a lipid layer, disruption of BBB by means of ultrasound technique or radiation therapy and use of nanomaterials are some methods to help drug delivery across BBB (Poovaiah et al., 2018). Among these methods, nanotechnology-based drug delivery is a quite new as compare to conventional methods and promising approach in the field of neuro disorders, as nanocarriers have been proved efficacious in effectively traversing the BBB or BCSFB and thus effectively delivering drugs within the CNS to the target site (Silva, 2005; Jain, 2007; Wong et al., 2012; Li et al., 2017). The Table 1 describes the few of the FDA approved nanodrugs available clinically for different indications (Ventola, 2017). The average size of human cells is 10–20 μm, where as the minimal diameter of blood capillaries are 6–9 μm, due to their nano size ranges nanomaterials get easily transported and internalizes by brain capillary endothelial cells via endocytosis and transcytosis mechanism of transport (Vilella et al., 2014). Different nanocarriers of particle size between 1 and 100 nm have been emerged by the development of nanotechnology, among which polymeric nanoparticles (PNPs), solid lipid nanoparticles (SLNPs), liposomes, and micelles made their debut as nanocarriers for treatment of various neurological disorders. But, nowadays, newer and more advanced nanosystems, such as dendrimers, nanoemulsions (Bonferoni et al., 2019), nanogels, nanosuspensions, and nanotubes, etc., which have shown great potential than previous delivery systems, developed by the nanotechnological approach (Rajadhyaksha et al., 2011; Wong et al., 2012; Ozkizilcik et al., 2017; Alexander et al., 2019). The selection of an appropriate nanocarrier system is a prerequisite for effective delivery of drug to CNS across BBB. The size, surface area, surface charge and morphology of nanocarriers have a remarkable impact on their passage across CNS barriers. The nanocarriers used in CNS drug delivery should have optimum size, surface area, surface charge and should be biodegradable, non-toxic, biocompatible, cost-effective and site-specific (Chakraborty et al., 2017). Various nanodrug system such as polymers, micelles, liposomes, dendrimers, nanocrystals, SLNPs have been used to improve efficacy, safety physiological properties pharmacokinetics, and pharmacodynamics of drugs (Figure 2) (Shegokar and Müller, 2010; Chan and Kwok, 2011; Gao et al., 2012; Thomas et al., 2013). The major targets in the development of nanomedicine are:

**TABLE 1:** Clinically available FDA-approved nanoformulated drugs (Sainz et al., 2015; Bobo et al., 2016; Caster et al., 2017; Centerwatch, 2017; Flexion Therapeutics Inc, 2017; Food and Drug Administration, 2017; Ventola, 2017).

Generic name	Trade name (Manufacturer)	Disease	Advantages
**Polymer NPs**			
Copaxone (Teva)	Glatimer acetate	Multiple sclerosis	Controlled clearance
Eligard (Tolmar)	Leuprolide acetate and polymer	Prostate cancer	Extended circulation time, controlled payload delivery
Adynovate (Shire)	Antihemophilic factor (recombinant), pegylated	Hemophilia	Better protein stability, longer half-life
Cimzia (UCB)	Certolizumab pegol	Crohn’s disease, rheumatoid arthritis, psoriatic arthritis, and ankylosing spondylitis	Extended circulation time, greater stability *in vivo*
Mircera (Vifor)	Methoxy polyethylene glycol-epoetin beta	Anemia associated with CKD	High aptamer stability
Neulasta (Amgen)	Pegfilgrastim	Chemotherapy-induced neutropenia	High protein stability
Krystexxa (Horizon)	Pegloticase	Chronic gout	High protein stability
Macugen (Bausch and Lomb)	Pegaptinib	Neovascular AMD	High aptamer stability
Adagen (Leadiant Biosciences)	Pegademase bovine	SCID	Longer circulation time, decreased immunogenicity
Oncaspar (Baxalta United States)	Pegaspargase	ALL	High protein stability
Pegasys (Genentech)	Pegylated IFN alpha-2a	Hepatitis B, hepatitis C	High protein stability
PegIntron (Merck)	Pegylated IFN alpha-2b	Hepatitis C	Greater protein stability
Somavert (Pfizer)	Pegvisomant	Acromegaly	High protein stability
Zilretta (Flexion Therapeutics)	Triamcinolone acetonide ER injectable suspension	Osteoarthritis knee pain	Extended release
Plegridy (Biogen)	Pegylated IFN beta-1a	Multiple sclerosis	High protein stability
Rebinyn (Novo Nordisk)	Coagulation factor IX (recombinant), glycopegylated	Hemophilia B	Prolonged half-life, higher drug levels between infusions
Renvela (Genzyme); and Renagel (Genzyme)	Sevelamer carbonate; and Sevelamer HCl	CKD	Prolonged circulation time and therapeutic delivery
**Liposome NPs**			
DepoDur (Pacira Pharmaceuticals)	Liposomal morphine sulfate	Postoperative analgesia	Prolonged release
Marqibo (Spectrum Pharmaceuticals)	Liposomal vincristine	ALL	High delivery to tumor site, decreased systemic toxicity
Onivyde (Ipsen Biopharmaceuticals)	Liposomal irinotecan	Pancreatic cancer	High delivery to tumor site, decreased systemic toxicity
Curosurf (Chiesi United States)	Poractant alfa	Respiratory distress syndrome	High delivery with low volume, decreased toxicity
Doxil (Janssen)	Doxorubicin HCl liposome injection	Karposi’s sarcoma, ovarian cancer, multiple myeloma	High delivery to disease site, less systemic toxicity
Abelcet (Sigma-Tau)	Liposomal amphotericin B	Lipid complex fungal infections	Reduced toxicity
AmBIsome (Gilead Sciences) B	Liposomal amphotericin	Fungal/protozoal infections	Reduced nephrotoxicity
Visudyne (Bausch and Lomb)	Liposomal verteporfin	Wet AMD, ocular histoplasmosis, myopia	Improved delivery to site of diseased vessels
Vyxeos (Jazz Pharmaceuticals)	Liposomal daunorubicin and cytarabine	AML, AML with myelody splasiarelated changes	Enhanced efficacy through synergistic delivery of
**Micelle NPs**			
Estrasorb (Novavax)	Micellar estradiol	Vasomotor symptoms in menopause	Controlled delivery
**Inorganic NPs**			
Ferrlecit (Sanofi-Aventis)	Sodium ferric gluconate complex in sucrose injection	Iron deficiency in CKD	Increased dose
Infed (Actavis Pharma)	Iron dextran	Iron deficiency in CKD	Increased dose
Venofer (American Regent)	Iron sucrose	Iron deficiency in CKD	Increased dose
Dexferrum (American Regent)	Iron dextran	Iron deficiency in CKD	Increased dose
Feraheme (AMAG Pharmaceuticals)	Ferumoxytol	Iron deficiency in CKD	Prolonged, steady release with less frequent dosing
**Protein NPs**			
Ontak (Eisai)	Denileukin diftitox	Cutaneous T-cell lymphoma	Targeted T-cell specificity, lysosomal escape
Abraxane (Celgene)	Albumin-bound paclitaxel	Breast cancer, NSCLC, pancreatic cancer	Greater solubility, increased delivery to tumor
**Nanocrystal NPs**			
Tricor (AbbVie)	Fenofibrate	Hyperlipidemia	Increased bioavailability simplifies administration
Vitoss (Stryker)	Calcium phosphate	Bone substitute	Mimics bone structure
Zanaflex (Acorda)	Tizanidine HCl	Muscle relaxant	High drug loading and bioavailability
Avinza (Pfizer)	Morphine sulfate	Psychostimulant	High drug loading and bioavailability, ER
EquivaBone (Zimmer Biomet)	Hydroxyapatite	Bone substitute	Mimics bone structure
Emend (Merck)	Aprepitant	Antiemetic	Absorption and bioavailability increases
Focalin (Novartis)	Dexamethylphenidate HCl	Psychostimulant	Higher drug loading and bioavailability
Megace ES (Par Pharmaceuticals)	Megestrol acetate	Antianorexic	Lower dosing
Invega Sustenna (Janssen)	Paliperidone palmitate	Schizophrenia, schizoaffective disorder	Slow release of injectable low-solubility drug
NanOss (RTI Surgical)	Hydroxyapatite	Bone substitute	Mimics bone structure
Ostim (Heraeus Kulzer)	Hydroxyapatite	Bone substitute	Mimics bone structure
OsSatura (IsoTis Orthobiologics)	Hydroxyapatite	Bone substitute	Mimics bone structure
Rapamune (Wyeth Pharmaceuticals)	Sirolimus	Immuno-suppressant	Better bioavailability
Ritalin LA (Novartis)	Methylphenidate HCl	Psychostimulant	Higher drug loading and bioavailability
Ryanodex (Eagle Pharmaceuticals)	Dantrolene sodium	Malignant hypothermia	More rapid rate of administration at higher doses
**Inorganic NPs**			
Dexferrum (American Regent)	Iron dextran	Iron deficiency in CKD	Increased dose
Feraheme (AMAG Pharmaceuticals)	Ferumoxytol	Iron deficiency in CKD	Prolonged, steady release with less frequent dosing
Ferrlecit (Sanofi-Aventis)	Sodium ferric gluconate complex in sucrose injection	Iron deficiency in CKD	Improved dose
Infed (Actavis Pharma)	Iron dextran	Iron deficiency in CKD	Improved dose
Venofer (American Regent)	Iron sucrose	Iron deficiency in CKD	Improved dose
**Protein NPs**			
Abraxane (Celgene)	Albumin-bound paclitaxel	Breast cancer, NSCLC, pancreatic cancer	Better solubility, increased delivery to tumor sites
Ontak (Eisai)	Denileukin diftitox	Cutaneous T-cell lymphoma	Targeted T-cell specificity, lysosomal escape

*ALL, acute lymphoblastic leukemia; AMD, age-related macular degeneration; AML, acute myeloid leukemia; CKD, chronic kidney disease; ER, extended release; HCl, hydrochloride; IFN, interferon; NP, nanoparticle; NSCLC, non–small-cell lung cancer; SCID, severe combined immunodeficiency disease.*

(a) Safety, and high efficacy.
(b) Drug targeting to specific sites to reduce off target toxicity.
(c) Improved pharmacokinetic behavior by sustained drug release with wide safety margin.

Although it is quite difficult to say which nanoparticulate drug delivery system is having more potential and safe among listed nanocarriers such as polymers, micelles, liposomes, dendrimers, nanocrystals, SLNPs, the size, shape, composition, surface charge, monomers molar ratio, solubility of drug, their physiochemical properties and further their release in different acidic and basic environment inside cells, plays very important role in pharmacokinetics of each type of nanocarriers. Tables 2, 3 describes pharmacokinetics of each type of nanocarriers and their biocompatibility, respectively.

**TABLE 2:** Different nanoformulations with their biopharmaceutical properties.

Formulation type	Drug name	Route of administration	*in vivo* (Pharmacokinetics/Pharmacodynamics)	References
**Nanocrystals**				
	Fenofibrate	Oral	Enhanced oral bioavailability	Hanafy et al., 2007
	Megestrol acetate	Oral	Enhanced oral bioavailability	Sylvestre et al., 2011
	Nitrendipine	Oral	Enhanced oral bioavailability and hepatoprotection	Xia et al., 2010
	Nobiletin	Oral	Enhanced oral bioavailability and rapid absorption	Onoue et al., 2013
	Tranilast	Pulmonary Oral	Better anti-inflammatory effects in lung Enhanced bioavailability and rapid absorption	Kawabata et al., 2010; Onoue et al., 2011a
	Carbendazim	Oral	Enhanced oral bioavailability	Jia et al., 2003
	Cilostazol	Oral	Enhanced oral bioavailability	Jinno et al., 2006
	Curcumin	Oral	Improved oral bioavailability	Onoue et al., 2010
	Danazol	Oral	Improved oral bioavailability	Wu and Benet, 2005
**Solid lipid nanoparticles**				
Solid-in-oil nanosuspensions	Diclofenac Na	Dermal	Improved percutaneous absorption	Piao et al., 2008
Lectin-modified solid lipid nanoparticles	Insulin	Oral	Superior oral bioavailability	Zhang et al., 2006
Solid lipid nanoparticles	Lidocaine	Dermal	Controlled dermal permeation and duration of action	Pathak and Nagarsenker, 2009
Solid lipid nanoparticles	Azidothymidine	IV	Improved permeability and brain retention	Reddy et al., 2004
Solid lipid nanoparticles	Clozapine	IV	Improved systemic exposure, decreased clearance	Manjunath and Venkateswarlu, 2005
**Micelles**				
Block copolymeric micelles	Pilocarpine	Ocular	Improved miotic activity	Matsumura et al., 2004
Self-micellizing solid dispersion	Tranilast	Oral	Enhanced oral bioavailability	Onoue et al., 2013
Block copolymeric micelles	Camptothecin	IV	Extended systemic exposure	Watanabe et al., 2006
Block copolymeric micelles	Doxorubicin	IV	Improved systemic exposure, decreased clearance	Pepic et al., 2004
Block copolymeric micelles	Paclitaxel		Enhanced systemic exposure, decreased clearance	Kato et al., 2012
**Polymeric nanoparticles**				
PLGA and alginate nanoparticles	Clotrimazole/econazole	Oral	Enhanced oral bioavailability	Pandey et al., 2005
PLA-PEG nanoparticles	Docetaxel	IV	Extended half-life, enhanced antitumor effect	Hrkach et al., 2012
PLGA nanoparticles	Doxorubicin	IV, IP	Extended half-life, Reduced distribution to heart	Reddy et al., 2004
PLGA nanoparticles	Rifampicin	Oral	Improved oral bioavailability	Sharma et al., 2004
Chitosan analog nanoparticles	siRNA	Oral	Improved systemic distribution and gene silencing	Zhang et al., 2012
Albumin nanoparticles	Paclitaxel	IV	Low inter-/intrapatient variability, tumor targeting	
Hydrogel nanoparticles	Insulin	Oral	Improved oral bioavailability	Chaturvedi et al., 2013
PLGA nanoparticles	Glucagon	Pulmonary	Extended half-life and enhanced bioavailability	Onoue et al., 2011b
PLGA nanoparticles	VIP derivative	Pulmonary	Enhanced anti-inflammatory effects	Onoue et al., 2012
Ethyl cellulose/casein nanoparticles	Celecoxib	Oral	Enhanced oral bioavailability	Morgen et al., 2012
**Emulsion**				
Self-emulsifying drug delivery system	Cinnarizine	Oral	Improved oral bioavailability	Larsen et al., 2013
Self-emulsifying drug delivery system	Coenzyme Q_10_	Oral	Improved oral bioavailability	Onoue et al., 2012
Self-emulsifying drug delivery system	Cyclosporin A	Oral	Improved oral bioavailability	Strickley, 2004
Self-emulsifying drug delivery system	Simvastatin	Oral	Improved oral bioavailability	Thomas et al., 2012
**Dendrimers**				
Polylysine dendrimer	Doxorubicin	IV	Long-lasting systemic exposure, enhanced accumulation in tumor tissues	Kaminskas et al., 2012
Poly (amidoamine) dendrimer	Flurbiprofen	IV	High distribution and retention in site of inflammation	Asthana et al., 2005
PEGylated polylysine dendrimer	Methotrexate	IV	Prolonged systemic exposure	Kaminskas et al., 2011
Lactoferrin-conjugated dendrimer	Methotrexate	IV	Enhanced accumulation in lung	Kurmi et al., 2011
Poly (amidoamine) dendrimer	Piroxam	IV	Extended systemic exposure	Prajapati et al., 2009
**Liposomes**				
Liposome (PC/Chol)	O-palmitoyl tilisolol	IV	High distribution in neoplastic tissue	Kawakami et al., 2001
Liposome (PC/PG)	Paclitaxel	IV	Prolonged systemic exposure	Fetterly and Straubinger, 2003
Liposome (PC/Chol/10% DSPEPEG2000)	Prednisolone	IV	Increased and prolonged systemic exposure	Teshima et al., 2006
Liposome (Phospholipid/Chol)	Amikacin	IV	Extended half-life of the drug in vitreous	Honda et al., 2013
Liposome (PC/Chol/DSPG)	Amphotericin B	IV	Augmented systemic exposure, decreased RES uptake	Tomii, 2002
Liposome (DSPC/DSPG/Chol)	Cytarabine/daunorubicin	IV	Decreased clearance	Feldman et al., 2012
Liposome, PEGylated liposome	Doxorubicin	IV	High distribution in neoplastic tissue	Zhang et al., 2008

*RES, reticuloendothelial system; siRNA, small interfering ribonucleic acid; VIP, vasoactive intestinal peptide; IP, intraperitoneal; IV, intravenous; PC, phosphatidylcholine; PG, phosphatidylglycerol; Chol, cholesterol; DSPC, 1,2-distearoyl-sn-glycero-3-phosphocholine; DSPE,1,2-distearoyl-sn-glycero-3-phosphoethanolamine;DSPG,1,2-distearoyl-snglycero-3-phosphoglycerol; PEG, polyethylene glycol; PLA, polylactic acid; PLGA, poly (lactic-co-glycolic acid).*

**TABLE 3:** Biopharmaceutical and safety profile of nanoformulations (Onoue et al., 2014).

Biopharmaceutical properties		Safety	
Advantages	Disadvantages	Advantages	Disadvantages
**Engineered Nanoparticles**			
Improved systemic exposure High retention in mucosal layer Several dosage routes available	Low sustained releasing potency	Decreased gastric irritancy of NSAIDs	Toxic risk due to high C_max_ cytotoxic potential
**Lipid Nanosystems**			
Biodegradable and metabolized Specific drug delivery Prolonged systemic exposure	Rapid clearance due to RES uptake Limited dosage route	Low toxicity Low antigenicity	Cytotoxicity depending on the surfactant used
**Micelles**			
High membrane permeability High solubilizing potency Improved systemic exposure	Low sustained releasing potency	Low immunogenicity	Toxic risk due to high C_max_ Cytotoxicity depending on used surfactant
**Polymeric Nanoparticles**			
Stable *in vivo* drug release Long duration of action	Need to avoid initial burst Limited dosage route	Low immunogenicity	Need to be removed surgically for non-degradable polymers
**Dendrimers**			
Controlled release High solubility Specific rug delivery	Limited dosage route	Low immunogenicity	Hemototoxicity

Targeted delivery of the drug is a promising approach to minimize the side effects of a therapeutic agent. Nanocarrier based brain targeted delivery can be accomplished via receptor-mediated, transporter-mediated and pharmacological disruption of BBB. Decorating the delivery system with BBB receptor ligands, coupling antibodies, peptides or aptamers with BBB receptor ligand delivery system, and transporter ligands coupled micelle delivery are some of the approaches for nanocarriers based brain targeting (Guo et al., 2012; Gao et al., 2013). In the current review, we provide an overview of different nanoformulations of neuroprotective agents, with particular emphasis on their usefulness in different neurological diseases and the opportunities for future research.

## Nano Approaches Toward CNS Drug Delivery

Nanotechnology is an innovative, promising and cutting edge approach for delivering neurotherapeutics across BBB. In the last few decades, nanomedicines have shown great potential toward CNS drug delivery owing to its nanosize range, their unique physic-chemical properties and ability to exploit surface engineered biocompatible and biodegradable nanomaterials (Kaur et al., 2008). Nanotechnology-based approaches for site-specific delivery of therapeutics and other compounds across the BBB may potentially be engineered to carry out particular functions as needed. The drug, the pharmacologically active component to be delivered, itself constitutes one part of the nanoengineered complex while remaining complex is intended to accomplish other key functions such as encapsulating the active drug protection against enzymatic degradation, drug release at specific pH, ability to cross the BBB, and targeting specific brain cells (Silva, 2008). A wide range of pharmaceutical nanocarriers including liposomes, PNPs, SLNs, micelles, dendrimers, and some others have been developed (Rajadhyaksha et al., 2011; Wong et al., 2012; Ozkizilcik et al., 2017).

### Micelles

Micelles, the vesicles which is made up of amphiphilic surfactants (non-polymeric micelles) or amphiphillic copolymers (polymeric micelles) have recently fascinated the researchers as a novel drug carrier system to the CNS (Aliabadi and Lavasanifar, 2006; Torchilin, 2007). As compared to non-polymeric micelles, polymeric micelles are considered more stable having long duration of action and high biodistribution (Ozkizilcik et al., 2017). They have a core-shell structural design with size ranging from 10 to 100 nm consisting outer hydrophilic environment mostly made up of polyethylene glycol (PEG) and inner hydrophobic core synthesized by means of molecules such as polycaprolactone, polypropylene glycols, phospholipids, and fatty acids, thus they allow loading of hydrophobic drugs (Torchilin, 2007). The external hydrophilic shell provides stability to micelles in an aqueous environment and prolongs their circulation time in bloodstream, thus protecting it from reticulo-endothelial system (RES) and further facilitate their accumulation in specific region having leaky vasculature (Park et al., 2008). The class of pluronic (also known as Poloxamers) block copolymers is of particular interest as they have an ability to hinder drug efflux transporters, for instance, inhibition of P-gp efflux transporters widely expressed on BBB and enhance drug shipment to the CNS (Batrakova and Kabanov, 2008). Moreover, it was demonstrated that they facilitate the brain delivery of low molecular mass drugs incorporated into them by escalating the drug solubility and stability in plasma.

Number of attempts has been made to transform the micelles in such a way that enhanced concentration of loaded drug can cross on another side of BBB easily. One such modification is attaching either polyclonal antibodies against brain-specific antigen, α2-glycoprotein, or insulin to target the receptor at the luminal side of BBB. In mice, the intravenous administration of these modified micelles after loading with a fluorescent dye or neuroleptic drug haloperidol, resulted in improved delivery of the fluorescent dye to the brain and drastic increase in the neuroleptic effect of haloperidol (Kabanov et al., 1989).

Another modification of the micelle system is direct conjugation of the drug molecule and targeting moiety to the amphiphilic segment. For instance, Zhang et al. (2012) studied transferrin-modified cyclo-(Arg-Gly-Asp-d-Phe-Lys)-Paclitaxel conjugate-loaded micelle and demonstrated the increased uptake by the brain microvascular endothelial cells *in vitro* in addition to the lengthened retention in glioma tumor *in vivo* and no considerable toxicity was noticed. Poly lactic-glycolic acid (PLGA) nanoparticles (NPs) coated with Chitosan oleate (CS-OA) which imparts positive surface charge and Chitosan Oleate Self-Assembled Polymeric Micelles based nanosystems were prepared and compared, for their interaction with cells i.e., Caco-2 and Hela cells. micelles and PLGA NPs, loaded with lipophillic model drug i.e., resveratrol, based on release profiles, TGA analysis and the cell line interaction results PLGA-CS-OA found to be more stable compared with polymeric micelles (Miele et al., 2019).

### Liposomes

Liposomes are considered as the first generation of the novel colloidal nanocarriers which were successfully proven themselves as a drug carrier system in the 70’s (Bawarski et al., 2008). These are small spherical vesicles composed of the hydrophilic compartment at the center enclosed by a single or multiple phospholipid bilayers due to which they morphologically resemble with the cell membrane and are used as a strategic approach for drug delivery, proteins and peptides (Martins et al., 2007; Wong et al., 2012; Ozkizilcik et al., 2017). They can be classified into three categories depending upon their size and number of bilayers: small unilamellar (10–50 nm), large unilamellar (50–1000 nm), and multilamellar (20–100 nm). These are reversible structures due to presence of non-covalent interactions such as van der walls forces and hydrogen bonding between molecules (Ramos-Cabrer and Campos, 2013).

Unmodified conventional liposomes have short circulation time in the body as they are quickly eliminated from the systemic circulation by the cells of RES and therefore various attempts have been made to develop long-circulating and targeted liposomes (Musacchio and Torchilin, 2011). Among such attempts, polyethylene glycol (PEG) coating on liposomes is a successful attempt to avoid RES recognition of nanocarrier system (Barenholz, 2012; Wong et al., 2012). Targeted delivery of PEG-modified liposomes to the brain can be facilitated by further modifications with various ligands like monoclonal antibodies (mAbs) against glial fibrillary acidic proteins, transferrin receptors (TRs) or human insulin receptors (Pardridge, 1999; Shah et al., 2013). Transferrin-conjugated liposomes have been demonstrated to preferentially deliver the payload like 5-fluorouracil to the brain facilitated by receptor-mediated endocytosis (Soni et al., 2008). Conjugation of prednisolone-loaded liposomes with mAbs that will be recognized by cell surface receptors in the targeted tissue called immunoliposomes, demonstrated further improvement in distribution of liposomes inside brain and high efficacy against experimental autoimmune encephalomyelitis (Schmidt et al., 2003). Immunoliposomes as nanocarrier systems for brain drug delivery also have some usage in gene therapy as demonstrated by TRsMAbs-targeted liposome conjugated with a plasmid for tyrosine hydroxylase in treating PD in a rat model (Zou et al., 2010). This approach has also been used for delivery of small interfering RNA (siRNA) against epithelial growth factor receptor (EGFR) and demonstrated the knock-down of EGFR expression and increased survival of mice implanted intracranially with brain tumors (Pardridge, 2007). Another approach to improve the efficiency of liposomes in crossing the barriers and increase therapeutic success is their modifications with cell penetrating peptides (CPP). For instance, specific ligand transferrin (T7) and non-specific cell penetrating peptide (TAT) conjugated doxorubicin encapsulated liposomes demonstrated high availability across BBB and specific cell targeting to the brain glioma (Zong et al., 2014). Recently nimodipine proliposomes, which form liposomal structure upon contact with water, augment the oral bioavailability of the drug (Sun et al., 2013). In another study, compounds like α-tocopherol (Toc) and omega3 fatty acid were loaded into liposomes with anti-Alzheimer drug tacrine for the treatment of AD with intranasal route (Corace et al., 2014).

### Polymeric Nanoparticles

Polymeric nanoparticles are solid colloidal dispersion of biodegradable and biocompatible polymers such as poly (alkylcyanoacrylate) (PACA), polyesters such as poly (lactide) (PLA), poly (D,L-lactide-co-glycolic acid) (PLGA), and several others such as natural proteins and polysaccharides with size range 10–100 nm (Kumari et al., 2010). They consists a core of dense polymer matrix to encapsulate the lipophillic drugs and a hydrophilic corona to provide steric stability to NPs. The drug to be delivered may be encapsulated, adsorbed or chemically linked to the surface of the NPs (Sahoo et al., 2017). The residence time of these NPs in systemic circulation can be increased by the surface modification either with physical adsorption or covalent binding of hydrophilic polymers such as PEGs and polysaccharides, whereas the inclusion of tissue-specific ligands facilitates targeted delivery to the brain (Mohamed and Van Der Walle, 2008; Wilson et al., 2008). It has been established that coating of poly (n-butylcyanoacrilate) (PBCA) NPs with 1% polysorbate 80 (PS80) amplified the concentration of rivastigmine or tacrine drug inside the brain as compared with free drug and selectively targeted to the CNS for AD reducing the hepatic or gastrointestinal side-effects coupled with conventional treatment approach (Wilson et al., 2008). Another study proved that dalargin containing PS80-coated PACA nanoparticles was capable to cross the BBB and produce its antinociceptive effect, after oral administration (Das and Lin, 2005). A possible mechanism of such delivery is adsorption of PS80-coated PACA nanoparticles on ApoE and B from the bloodstream upon intravenous injection followed by transcytosis across BBB using the low-density lipoprotein receptors (Kreuter, 2013). PLGA nanoparticles exhibits good carrier for delivering drugs across BBB. *In vivo* distribution of vanlafaxine loaded PLGA nanoparticles against depression was tested in C57/bl6 mice and in *vitro* BBB model using hCMEC/D3 cell. They observed that transferring receptor mediated PLGA nanoparticles have better biodistribution in brain via intranasal administration and not affected by P-gP pump efflux enhacing functionalized nanoparticles concentration in the basolateral side after 24 h via receptor mediated endocytosis (Cayero-Otero et al., 2019).

### Solid-Lipid Nanoparticles

Solid-lipid nanoparticles (SLNs) are attracting major attention as novel drug carriers nowadays and are at the forefront of the rapidly emerging nano-delivery system (Kreuter et al., 2002). These are aqueous colloidal nanocarriers system which are composed of physiological lipid (triglycerides, fatty acids, steroids, and waxes, etc.), dispersed in water or in an aqueous surfactant solution and possess the ability to get solidify upon cooling (Barchet and Amiji, 2009; Mukherjee et al., 2009). A number of efforts have been made to increase the drug loading capacity and long-term stability of SLNs, one such effort being the development of nanostructured lipid carriers (NLCs) by amalgamation of spatially dissimilar lipids or blending solid lipids with liquid lipids. Modification of SLNs with PEG increases penetration to BBB and enhances drug delivery to the CNS as demonstrated by a comparative study of SLNs and PEG-modified SLNs loaded with antitumor drugs like camptothecin and doxorubicin (Blasi et al., 2007; Wong et al., 2007). SLNs are preferred more over PNPs due to numerous advantages such as low intrinsic cytotoxicity, physical stability, shielding of labile drugs from degradation, controlled release which provide them potentiality to be used as a brain drug delivery system, especially for brain tumors (Laquintana et al., 2009). The possible mechanism of their delivery across BBB may be endocytosis, transcytosis which takes place in the endothelial cells lining the blood capillaries in brain or permeation through the tight junctions between endothelial cells. Furthermore, the adsorption of a plasmatic protein such as the apolipoprotein E, onto SLNs surface could facilitate its uptake into brain mediated by adherence to the endothelial cells of the BBB (Kreuter et al., 2002; Reddy and Venkateswarlu, 2004; Blasi et al., 2007). The above approach has been used for the encapsulation of a broad range of drugs to achieve target-specific delivery of drugs across BBB. Sterylamine-based SLNs containing clozapine, an antipsychotic drug have been fabricated and demonstrated to deliver the drug successfully inside the brain after intravenous and intraduodenal administration (Manjunath and Venkateswarlu, 2005). Other examples of drug loaded SLNs include quercetin loaded SLNs to treat AD (Dhawan et al., 2011), atazanavir loaded SLNs for treatment of HIV-encephalitis (Chattopadhyay et al., 2008). Recently, it was observed that riluzole-loaded SLNs have greater efficacy than free riluzole in amyotrophic lateral sclerosis (ALS) rat model developed by immunization using the experimental allergic encephalomyelitis (Bondì et al., 2010).

### Nanoemulsions

Nanoemulsions are either oil-in-water (O/W) or water-in-oil (W/O) colloidal particulate systems that are composed of edible oils, surface-active agents (surfactants) and water, having a size range 100–500 nm (Sarker, 2005; Lovelyn and Attama, 2011). Recently, their use as drug delivery system has been promoted heavily to tackle several problems associated with conventional delivery systems such as low bioavailability, poor targetability, and penetrability across BBB (Lovelyn and Attama, 2011). Various oils and surface modifiers that are used in preparation are the key determinants of the versatility of nanoemulsions (Sarker, 2005). For instance, fabrication of nanoemulsions with oils that are rich in omega-3 polyunsaturated fatty acids (PUFA) imparts specific properties to nanocarriers for overcoming biological barrier, including the BBB, therefore help in achieving the rapid distribution of drugs to peripheral sites, especially the brain (Shah et al., 2013). The nanoemulsion system containing pine-nut oil significantly increased the oral bioavailability of paclitaxel (Tiwari and Amiji, 2006).

### Dendrimers

Dendrimers are the emerging polymeric structures that are known for their defined structures, and versatility in drug delivery. They have 3-D symmetrical architecture possessing an inner core from which a number of hyper-branches, known as “generations,” having various chemical functional groups at the peripheral terminal surface arise due to which they can be easily functionalized with different ligands (Tripathy and Das, 2013; Xu et al., 2013; Leiro et al., 2018; Dias et al., 2020). Depending upon the generations, complexity and building block materials, a wide range of dendrimers has been developed among which polyamidoamine (PAMAM), polypropylene imine (PPI), and polylysine dendrimers are of intense interest for both hydrophobic and hydrophilic drug molecules delivery. The high molecular weight hydrophilic/hydrophobic entities either physically entrap/conjugate by host-guest interactions or covalently bound with peripheral functional groups of dendrimers to form a dendrimer-drug complex (Tripathy and Das, 2013). Although, dendrimers have wide application in drug delivery, yet their use in biological systems is limited due to complex synthetic procedures and toxicity issues (Kaminskas et al., 2011; Tripathy and Das, 2013).

**FIGURE 3:**
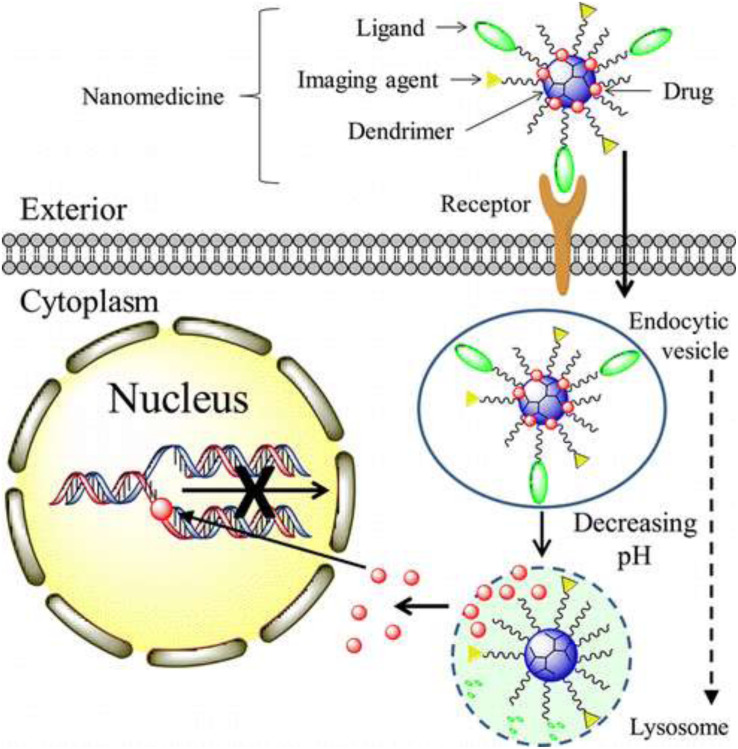
Mechanism of nanotherapeutics, such as methotrexate and doxorubicin, delivery by dendrimers for brain tumor. Reproduced with permission from Xu et al. (2013). Copyright @ 2014, American Chemical Society.

**FIGURE 4:**
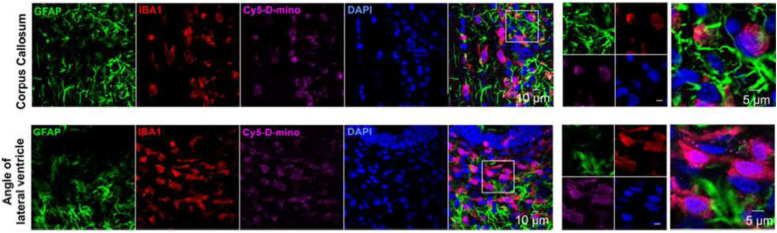
*In vivo* cellular localization of Cy5-D-mino. The endotoxin kits with Cerebral Palsy (*n* = 3) received Cy5-Dmino (55 mg/kg, i.v) on Post Natal Day 1 and sacrificed 24 h post-injection. Brain slices contain Cy5-D-mino (magenta) were co-stained with GFAP (astrocyte marker, green)/IBA1 (microglial marker, red)/DAPI (blue). Cy5-D-mino is mainly co-localized with activated microglia at the corpus callosum and angle of lateral ventricle. The images on the right panels are the higher magnification of the region of interest marked with the boxes on the left panels. Reproduced with permission from Sharma et al. (2017). Copyright @ 2017, American Chemical Society.

Nowadays, as a drug carrier, dendrimers are given more preference over traditional polymers due to various features such as high aqueous solubility, biocompatibility, polyvalency, and precise molecular weight which make them amenable for targeted drug delivery in the brain (Duncan and Izzo, 2005; Tomalia, 2005). They have shown potential as carriers of drugs, therapeutic nucleic acids, peptides and proteins, contrast agents for CNS disorders treatment, imaging, and diagnosis (Xu et al., 2014; Figure 3). Aso et al. (2019) synthesized Poly (propylene imine) dendrimers core and maltose-histidine shell (G4HisMal) and check their biocompatibility and ability to penetrate BBB, they were tested it against AD and found their high ability to cross BBB with significant biocompatibility showing memory protection in transgenic mice model of Alzheimer disease via synapse protection. The *in vitro* efficacy and *in vivo* targeting capability of hydroxyl-G6 PAMAM dendrimer–9-amino-minocycline conjugate (D-mino) was checked, additionally D-mino was assessed for anti-inflammatory and antioxidant activity in lipopolysaccharides-activated murine microglial cells. The rapid uptake, decreases inflammatory cytokines and nitric oxide levels suggested that dendrimers formulation are having high efficacy as compare to free drug, further it was observed that fluorescently labeled dendrimer conjugate (Cy5–D-mino) crosses the blood brain barrier much efficiently than free drug (Sharma et al., 2017; Figure 4).

Targeted delivery by dendrimers can be achieve by attaching target ligands such as transferrin family ligands having an affinity for TRs, mAbs against specific receptors, lectins such as wheat germ agglutinin (WGA), etc (Tomalia, 2005). The dendritic approach has been applied to improve the solubility and oral absorption of various drugs such as camptothecin (CPT), by conjugating drugs with PAMAM dendrimers (Sadekar et al., 2013). Recently, it was demonstrated that cisaconityl linkage, an acid-sensitive linkage, in PAMAM-PEG-Doxorubicin (DOX) conjugates followed an acid-triggered release kinetics which gradually increases with increasing degree of PEGylation and simultaneously imparts following properties to the conjugates: reduced hepatic and splenic accumulation, prolonged circulation time, and enhanced tumor buildup of the conjugates (Zhu et al., 2010a, b).

## Mechanism of Action of Drug Release

The physiochemical properties of drug loaded nanocarrier like hydrophilicity, surface charge and targeting ligands of nano carrier is the deciding factor for the adsorption on the brain capillary endothelial cells. Surface charge like positive nature of the nanomaterials interacts with the negative surface charge of endothelial cells of brain, due to electrostatic interactions between carrier and cells, secondly lipophillic nature of nanocarriers also enhance and facilitates adsorption process (Markoutsa et al., 2011; Lien et al., 2012). These approaches reduces the clearance of nanocarrier by the fixed macrophages of the mononuclear phagocytic system (MPS), Once, the nanomaterials get absorbs either through targeting low density lipoprotein receptors present on the microvessel brain capillary endothelial cells (Kim et al., 2007) by means of normal endocytosis and transcytosis, desorption occurs and reenter into the blood stream, where drug loaded nanocarrier releases its encapsulated or adsorbed drug on the surface of blood brain barrier and further diffuses into brain parenchyma. The average size of human cells is 10–20 μm, where as the minimal diameter of blood capillaries are 6–9 μm, due to their nano size ranges nanomaterials get easily transported and internalizes by brain capillary endothelial cells via endocytosis and transcytosis mechanism of transport (Vilella et al., 2014). The adsorbed drug–carrier conjugates is endocytosized by the cells, at times followed by exocytosis, and further penetration of the nano carrier into the cells or brain parenchyma occurs.

The mechanism of drug delivery by nanotechnology is not still well-explored, however, various suggestions have been put forward to suggest possible mechanisms. Though the uptake of nanocarriers along with drugs into the brain has been proposed to occur by following six mechanisms (Kreuter, 2001, 2013; Wohlfart et al., 2012):

(i) Increased transport across the endothelial cell layer facilitated by higher concentration gradient due to increased retention of the nanocarriers in the brain blood capillaries with an adsorption to the capillary walls. Since the diffusing drug can be effluxes out by various transporters such as P-gp, it is not a good mechanism to achieve a sufficient amount of drug inside the brain to elicit a relevant pharmacological response.
(ii) Inhibition of the efflux system particularly P-gp.
(iii) Toxicity to the brain vasculature.
(iv) Solubilization of the endothelial cell membrane lipids due to general surfactant effect followed by membrane fluidization and enhanced drug permeability across the BBB.
(v) Permeation of nanocarrier system through the tight junctions after the opening of them.
(vi) Endocytosis and transcytosis phenomenon through the endothelial cell layer.

Since the entry of foreign entities in CNS is strictly prohibited by BBB, only selective one gets access to the brain. They get entry into the brain either by passive, gradient-dependent (passive targeting) or active, energy-dependent (active targeting) pathways. As the passive movement of water solutes through the clefts between adjacent cells (paracellular transport) is negligible at physiological conditions due to the presence of tight junctions as well as the early phases of neurological disease do not involve any defects in BBB to allow drug transport, implication of this transportation pathway for delivery of drug-loaded nanocarrier is not a successful approach. As an alternative, consideration should be given to the transportation of nanocarriers per se through the endothelial cells (transcellular transport). The nanocarriers of size less than 500 Da having the ability to absorb inside cells are suitable for transcellular transportation (Georgieva et al., 2014). Transcytosis process especially the receptor-mediated transcytosis across BBB is an attractive strategy to deliver drugs into CNS. It requires vector for the delivery of nanocarrier between the apical and basolateral surface in polarized cells. Receptor-mediated internalization of cargo followed by vesicular transport is a suitable option as nano-delivery systems of size 100–200 nm range can be easily accommodated in these transport vesicles. Three distinct steps are involved in overall transcytosis process: endocytosis of the nanocarrier at the plasma membrane, trafficking of intracellular vesicles toward the opposite surface followed by exocytosis (Rappoport, 2008; Pardridge, 2012; Georgieva et al., 2014).

Internalization via phagocytic mechanism occurs by various pathways among which clathrin-dependent and caveolin-dependent pathways are the major one:

(i) Clathrin-mediated endocytosis happens in all mammalian cells. As the nanocarrier binds with a specific receptor on the cell membrane, it triggers the polymerization of clathrin-1, a cytosolic protein underneath the plasma membrane forming an inward budding leading to the internalization of cargo (Rappoport, 2008). After wrapping of the nano-delivery system, the GTPase activity of dynamin pinches off the inward budding resulting into the formation of clathrin-coated vesicles (Pucadyil and Schmid, 2009). The clathrin coat shed off during the movement of vesicles inside cytosol with the help of energy provided by actin, leading to the formation of early endosomes as detected by early endosome antigen-1 (EEA-1), a typical marker. The early endosome delivers their content to late endosomes and finally to the lysosomes where it is degraded off. The compartmental pH gradually falls during the transition of late endosomes to the lysosomes, which triggers the release of drug from nano-vehicle and eventually the drug is released at the desired site (Mellman, 1992; Georgieva et al., 2014).
(ii) Another well known pathway for delivery of nanocarriers inside the brain is caveolar pathway. This pathway is similar to the clathrin-mediated pathway in most of the aspects but there is possibility to escape lysosomal delivery which is a key factor that makes it different from the clathrin-mediated pathway (Mcintosh et al., 2002). For the same difference, this pathway augments the delivery of drugs at the targeted site and therefore improve the therapeutic value of drugs, thus targeting receptors associated with caveolae may prove good approach for transcellular delivery of the nanocarriers. Caveolae are flask-shaped plasma membrane invaginations and caveolin protein, present in three isoforms in mammalian cells: caveolin-1, caveolin-2, and caveolin-3 play a dominating role in this transportation pathway (Kou et al., 2013). As soon as the nanocarriers binds to the caveolar receptor, it is internalized forming a vesicular structure known as cavicle, by the involvement of dynamin in a similar way as in clathrin-mediated pathway. The cavicle moves inside the cytosol with the help of energy derived from actin and get fused with caveosomes which have neutral pH and then head toward the endoplasmic reticulum (ER). It is considered that nanocarrier from ER penetrates into the cytosol and finally gain access to the nucleus through the nuclear pore complex (Kasamatsu and Nakanishi, 1998; Parton and Simons, 2007; Kou et al., 2013).

## Applications of Nanotechnology in CNS Disorders

**FIGURE 5:**
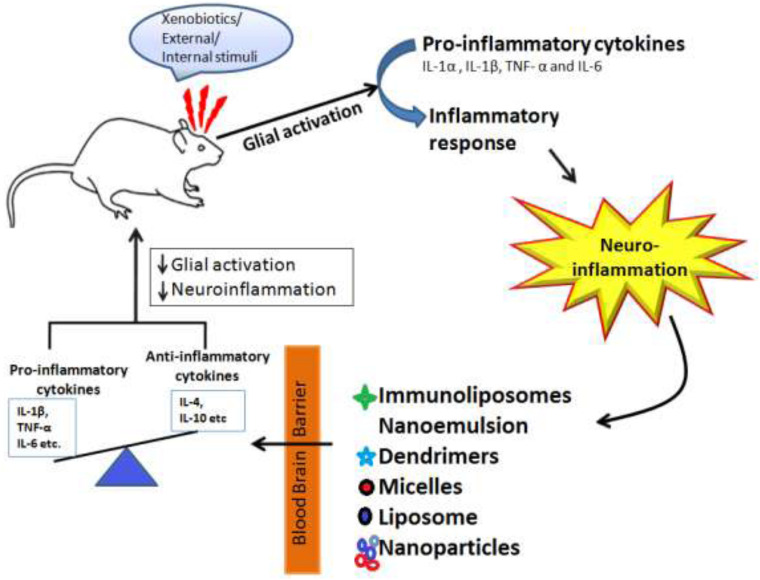
Schematic representation of nanotechnology-based therapy for CNS disorders.

Nanotechnology has revolutionized the field of treating various neurological disorders and has provided a number of new approaches that have shown potential for treating neurodegenerative disorders like AD, PD, stroke, epilepsy, HD, and brain tumor. Molecules are nano-engineered so that they have an ability to traverse the BBB, target the specific cell or signaling pathway, act as a carrier for gene delivery. Besides delivery of therapeutic drugs, nanotechnology has also gained the interest of researchers for delivery of radiocontrast agents, imaging agents for diagnosis purpose. Figure 5, is the schematic representation of nanotechnology-based therapy for CNS disorders depicted that when an external stumuli or in diseased conditions glial activation occurs which leads to neuroinflammation and leads to imbalance between pro-inflammatory and (IL-1β, TNF-α, and IL-6) and anti-inflammatory cytokines (IL-4, IL-10, etc.), here the authors are showing through schematic representation that drugs loaded with nanoformulations such as immunoliposomes, dendrimers, micelles, liposomes, and nanoparticles due to their small size and specific targeting ability stabilizes and modulate the release of drugs to cross the blood brain barrier and restores the healthy conditions via reducing neuroinflammation and glial cells activation.

### Alzheimer’s Disease

Alzheimer’s disease is a slowly progressive neurodegenerative disorder, and is the main culprit for dementia syndrome. Pieces of evidence state that its incidence and prevalence rate is more common in elder persons (Sloane et al., 2002). Amyloid-β plaques and Tau protein hyper-phosphorylation are considered as hallmarks of AD (Kumar and Singh, 2015). The degeneration of nervous tissue in AD starts years back of the actual appearance of the symptoms of the disease. The conventional treatment approaches are not able for complete treatment of disease, thus the inability of orally administered drugs such as tacrine, rivastigmine, etc. to treat the disease, opens the door for the application of nanotechnology for AD treatment.

A wide range of nano-formulations has been fabricated to have a beneficial impact on AD patients. It was demonstrated that PEG stabilized nanomicelles composed of phospholipids inhibit Aβ aggregation and attenuate Aβ-induced neurotoxicity in SHSY-5Y human neuroblastoma cell line *in vitro* (Pai et al., 2006). The *in vitro* study demonstrated the ability of phytochemical curcumin to reduce Aβ oligomerization and cytotoxicity, but poor bioavailability was shown when injected into mice (Yang et al., 2005). The nanoliposomal formulation of curcumin enhanced its bioavailability without disturbing its ability of inhibiting Aβ aggregation (Taylor et al., 2011).

Since the increased level of metal ions, such as copper, also contributes in the pathology of AD, use of chelating agents is another approach for AD management (Lovell et al., 1998). Microemulsion nanoparticles loaded with copper chelator d-penicillamine were found to have capability of crossing the BBB and dissolving the pre-existing Aβ aggregates *in vitro* (Cui et al., 2005).

Oxidative damage, another key factor in the pathology of AD, suggests the applications of antioxidants in AD management. Fullerene derivatives, the potent free-radical scavengers, have been found to possess neuroprotective action against glutamate receptors induced excitotoxicity. On the other hand ability of fullerene for neuroprotection, *in vitro* as well as *in vivo*, against Aβ toxicity has not been proved, yet its ability to inhibit Aβ peptide fibrillization and prevention of Aβ-induced cognitive impairments after intraventricular administration suggests its beneficial role in AD treatment (Podolski et al., 2007).

Marked deficiency of acetylcholine (ACh) neurotransmitter is another important feature in the pathology of AD. Due to its faster decomposition in blood, the direct injection of free ACh is not effective for reversal of the imbalance in ACh. Thus, nanotechnological approach has been used for delivery of ACh in the brain to achieve balanced ACh level. The ACh loaded in carbon nanotubes have been found to restore the significant cognitive functions to pre-AD level in a kainic-acid induced mouse model as compared to free ACh (Yang et al., 2010).

### Parkinson’s Disease

Parkinson’s disease, illustrated by loss of dopaminergic neurons in the substantia nigra of the midbrain and the generation of α-synuclein aggregates (Lewy bodies), is the next most common neurodegenerative disorder worldwide after AD. Nowadays, it is considered that pathology of PD is not just limited to a particular part of the brain but it involves various other regions of the brain, neurotransmitters such as imbalance in ACh and dopamine, and protein aggregates other than Lewy bodies (Kalia and Lang, 2015). The manifestations of PD involve motor symptoms like tremor, speech, writing changes and non-motor symptoms like cognitive, behavioral, and autonomic changes.

The current medication treatments available for PD do not cure nor alleviate the progression of the disease but aim to provide the symptomatic relief to the patients, and beside this, the inability of available drugs to cross BBB is another challenge for treating PD. As a result, development of innovative and more effective therapy is the need of hour. This problem can be addressed by the nanotechnological approach, as it has shown great potential to reverse or limit the disease states, promote the functional regeneration of damaged neurons, provide neuroprotection and facilitate the delivery of drugs overcoming the BBB (Modi et al., 2010; Soursou et al., 2015).

Gene delivery approach has been intensely exploited in the perspective of PD. The conventional gene delivery studies involve viral vectors, but they were commonly associated with toxicity and immunogenicity problems while nanotechnological approach was found to be devoid of such type of problems (Witt and Marks, 2011). The PEG and polyethyleimine nanogels complexes with antisense oligonucleotides demonstrated the efficient crossing of BBB *in vitro* and also delivered the oligonucleotides to the brain, when injected intravenously, more efficaciously particularly when the gels were functionalized with insulin or Tf molecules (Vinogradov et al., 2004). In another study on 6-hydroxydopamine (6-OHDA) model of PD in rats, the striatal tyrosine hydroxylase enzyme activity was restored with reversal of motor impairment, after a single intravenous administration of tyrosine hydroxylase-encoding plasmids and Tf receptor antibody conjugated PEGylated liposomes (Zhang et al., 2003). Recent study has confirmed that nerve growth factor (NGF) bound PBCA nanoparticles (Kurakhmaeva et al., 2009) and L-Dopa encapsulated nanoparticles cross BBB and alleviate basic symptoms of PD (Mohanraj et al., 2013).

mPEG PLGA nanoparticles of size range 70 nm i.e., Schisantherin A (SA) was used against Parkinson diseases (PD) in zebrafish larvae where SA encapsuled in nanoparticles formulation that extended SA circulation in the blood stream and consequently an increased brain uptake and reported as potentially efficacious for the treatment of PD. It was found that nanoparticulate delivery of SA was much more effective than SA suspension alone. In addition, the SA-NPs exerted strong neuroprotective effects in zebrafish and cell culture model of PD (Chen et al., 2017).

### Huntington Disease

Huntington disease (HD) is described by preferential loss of neurons in striatum and other brain regions leading to progressive motor, cognitive, and psychiatric manifestations. It occurs due to monogenic mutation in the exon 1 of huntingtin gene which leads to polyglutamine (poly Q) expansion and causes misfolding and aggregation of huntingtin protein (HTT) in the brain (Bates et al., 2015). Several studies confirm the involvement of astrocytes in HD. Brains of HD patients and mouse models of HD demonstrates accumulation of mutant HTT in striatal astrocytes which ultimately leads to age-dependent HD-like pathology and premature mortality (Bradford et al., 2009). Symptomatic and protective treatment strategies are available for HD but none of them are efficacious to completely cure the disease. Currently, tetrabenazine is FDA approved drug for the symptomatic treatment of HD.

A possible linkage between oxidative stress and neurodegenerative disorders such as HD has been shown in literature (Kim et al., 2015; Liu et al., 2017). Therefore, antioxidant therapy can be employed in HD to prevent oxidative stress. Fullerenols have ability to clear free radicals and reduce oxidative burden to cell. Jin et al reported their antagonistic behavior on glutamate receptors and therefore they can be implicated for neuroprotective use (Jin et al., 2000).

Nitrendipine is a calcium channel blocker that declines the incidences of dementia (upto 50%) in HD in a period of 2 years. Being hydrophilic, this drug has permeability issues and thus poorly crosses BBB. In a study, SLNs of nitrendipine were prepared and a comparison in the uptake of bulk drug and nanoformulation was done. The results demonstrated the higher uptake of drug when it was encapsulated in SLNs (Manjunath and Venkateswarlu, 2006). In another study, short-interfering RNA (siRNA) encapsulated cyclodextrin nanoparticles were found capable to reduce expression of HTT mRNA both *in vivo* as well as *in vitro* (Godinho et al., 2013).

### Multiple Sclerosis

Multiple sclerosis (MS) is a progressive autoimmune and inflammatory disease. It is a multifactorial disease in which body’s immune cells attack on the nervous system. It involves various pathogenic mechanisms leading to demyelination of myelinated axons which results into slow conduction of nerve signal. There are evidences which suggest mutual interplay of microglial, astrocyte and T-cells beside demyelination (Khare et al., 2014; Ojha and Kumar, 2018). It is diagnosed on the basis of clinical findings, magnetic resonance imaging (MRI) and examination of Cerebrospinal fluid (CSF) (Khare et al., 2014). The prevalence rate of MS ranges 2–150 per 100,000 people and females are more susceptible than males (Jones et al., 2008). MS attack disrupt BBB in the section of brain or spinal cord from where peripheral T lymphocytes gain access to brain and attack the myelin gradually leading to demyelination (Zeis et al., 2009).

In past few years, advancements in nanotechnology have shown promising outcomes in diagnosis and treatment of MS. The theranostic approach has been found to possess potential applications in the field of medical care and cure of MS (Singh et al., 2012). The interaction of carbon nanotubes with stem cell provides new opportunities in nerve tissue engineering to explore and add to cell behavior (Huang et al., 2012). It provides alternative ways to treat MS pathology related to non-genetic manipulations of signaling pathways *in vivo* (Nunes et al., 2012). In a preclinical study, ciliary neurotrophic factor (CNTF) loaded microcapsules demonstrated *in situ* sustained delivery of CNTF upon encapsulation into polymers. This formulation was found to be devoid of any immune response and cytotoxic effect (Aebischer et al., 1996).

Beside role in therapeutics, nano-based approach also has implications in diagnosis of MS. DNA-carrier gold NPs based barcode assay is highly sensitive assay to detect biomarkers in CSF or diseased brain. Since radio diagnosis is a gold technique for detection of MS, this diagnostic assay may be highly useful in diagnosis of MS (Georganopoulou et al., 2005). All the recent developments made in nanotechnology opens a new door for diagnosis and treatment of MS pathologies.

### Tumor

Brain tumors, like other body parts tumors, may be benign, originating and residing within the brain, or metastatic, originating from a tumor outside the CNS, among which glioblastoma multiforme (GBM) – a malignant glioma, is the most prevalent. They are among the most challenging disease to treat due to poor prognosis, diagnosis, high recurrence rate, and availability of limited number of convenient methods to transport anti-cancer drugs across BBB in effective concentration (Sanai and Berger, 2008; Özdemir et al., 2017). Current treatments available are just palliative, involving united approaches of surgical debulking with radiotherapy and chemotherapy, which are not sufficient for the complete treatment, and also the reversal to the tumor state again is most common (Özdemir et al., 2017). The inability of conventional methods has provided a platform for the development of innovative nanotechnological approach as a novel imaging tool or to improve anticancer drug delivery into tumors while minimizing its distribution and toxicity in healthy tissues (Bidros and Vogelbaum, 2009).

Owing to the ability of glioma cells to aggressively infiltrate normal brain tissue and survive from the exposure to current adjuvant therapies, it is extremely important to construct specific targeted nanoplatforms capable of delivering imaging agents directly into invasive tumor cells. Owing to the ability of glioma cells to aggressively infiltrate normal brain tissue and survive from the exposure to current adjuvant therapies, it is extremely important to construct specific targeted nanoplatforms capable of delivering imaging agents directly into invasive tumor cells. Owing to the ability of glioma cells to aggressively infiltrate normal brain tissue and survive from the exposure to current adjuvant therapies, it is extremely important to construct specific targeted nanoplatforms capable of delivering imaging agents directly into invasive tumor cells.

Ability of glioma cells to infiltrate normal brain tissue and survive from the current adjuvant therapies opens the new door for employment of nano-based approach to deal with the glioma. Many researchers are trying to develop new nanotherapeutics approach to achieve a breakthrough in the treatment of gliomas. A broad range of nano-formulations for effective delivery of drugs across BBB has been developed and investigated (Li et al., 2014). Such nanoformulations include PBCA nanoparticles loaded with methotrexate (Gao and Jiang, 2006) and temozolamide (Tian et al., 2011), and have resulted in significantly increased intracerebral drug concentration as compared with free drugs. Similarly, the SLNs of etoposide (Lamprecht and Benoit, 2006) and paclitaxel (Garcion et al., 2006), *in vitro*, demonstrated the enhanced inhibitory effect on proliferation of glioma cell lines more efficiently than the free drug alone. Dendrimers have also been used to deliver antineoplastic treatments to the brain. Methotrexate conjugated to polyether-copolyester (PEPE) dendrimers demonstrated improved cytotoxicity against U87 and U343 cancer cell lines in culture and this nanoformulation was able to overcome acquired resistance to the drug, suggesting its role in combating resistance to the drug in gliomas (Dhanikula et al., 2008). Doxorubicin (DOX) loaded PEGylated PAMAM dendrimers demonstrated an extended therapeutic window by hindering C6 glioma spheroid proliferation and exhibited little cytotoxicity against brain microvascular endothelial cells *in vitro* (He et al., 2011). For imaging purpose, NPs as contrast agents offer detailed cellular and molecular imaging and thus provide information for efficient surgical removal of gliomas. Ultra small (less than 5 nm in diameter) gadolinium oxide crystals have been successfully used to label glioma cells GL-261 *in vivo* (Hernández-Pedro et al., 2013).

In a study, scientists encapsulated an antineoplastic drug in PEG-coated hexadecyl cyanoacrylate NPs and used against glioma. They observed controlled drug release kinetics along with higher diffusion of drug across BBB as compared to bulk drug in a rat model of gliosarcoma (Brigger et al., 2002). In an another study, epidermal growth factor receptors (EGFRs)-targeted therapy explored for treatment of glioma where angiopep-2 (A2)-modified cationic lipid-poly (lactic-co-glycolic acid) (PLGA) nanoparticle (A2-N) loaded Gefitinib (Ge), an EGFR tyrosine kinase inhibitor (TKI) and Golgi phosphoprotein 3 (GOLPH3)-siRNA were prepared. They demonstrated that Angiopep-2-modified cationic lipid polymer crosses the BBB. Gefitinib can inhibit EGFR signaling and block the autophosphorylation of critical tyrosine residues on EGFR whereas GOLPH3 siRNA downregulate GLOPH3 expression (Ye et al., 2019).

### Epilepsy

Epilepsy, a CNS disorder, is characterized by an abnormal increase in brain electrical activity that may be either limited to the focal area or spread throughout the brain, resulting in partial or generalized seizures, respectively (Jabir et al., 2015). The current treatment methods, having the aim of diminishing the seizure frequency and severity while producing the minimum toxic effects to the brain and other tissues of the body, are almost failures due to various hurdles, such as the inability of drugs to cross BBB, drug resistance and recurrence of disease after drug discontinuation. Various strategies such as nano-based approach, prodrugs, efflux pump inhibition, opening of BBB by hyperosmolar solution, direct drug delivery to the ventricles and cortex, gene therapy, have been developed for epilepsy treatment, but among these all, the nano-technological approach has shown great potential for overcoming all the major hurdles in epilepsy treatment including, crossing of BBB and targeted delivering of drugs at their therapeutic concentration (Bennewitz and Saltzman, 2009).

Solid lipid nanoparticles loaded with carbamazepine (Samia et al., 2012) and PLGA nanoparticles loaded with β-carotene (Yusuf et al., 2012) have been found to show more potential for anticonvulsant effect than nanoemulged loaded carbamazepine and polysorbate-80 coated PLGA nanoparticles, respectively. In a rat model, liposomal muscimol formulation (Kohane et al., 2002) has been reported to suppress focal seizures while producing minimal histological alterations, and amiloride loaded liposomes (Ali et al., 2007) demonstrated higher anticonvulsant potential in comparison to the free drug in a mice model. In another study on a rat model, it was found that the subcutaneous administration of the ethosuximide loaded chitosan nanocapsules decrease the spike wave discharge. Because of their ability to provide stable release of the drug, these nano-formulations can be fabricated as depot drug delivery systems for long-term use of antiepileptic drugs (Hsiao et al., 2012).

### Stroke

Stroke, an acute CNS disorder, is characterized by the disruption to the vasculature supplying the brain, resulting into sudden symptoms, within seconds to hours, which usually depends on the fraction of the brain involved and the severity. There are two main types of stroke: Ischemic stroke, contributing 87% of total stroke, comprising of lacunar, cardioembolic and cryptogenic stroke, and the hemorrhagic stroke, having 13% contribution to the total stroke, comprising of 10% intracerebral and 3% hemorrhagic stroke (Sacco et al., 2013). It is among the principal cause of mortality and morbidity, regardless of the availability of a number of treatment approaches, due to various challenges in drug treatment, which demands the development of new therapeutic approaches. The nanotechnological approach has shown great potential to overcome the major hurdles in stroke management and has provided a useful platform for the development of novel therapeutic methods for treatment (Sarmah et al., 2017).

Free radicals, which result in cellular and tissue damage, are considered as main contributing factors in the pathology of ischemic brain disease. The role of this contributing factor can be limited by the use of nano-therapeutic approach. Cerium oxide nanoparticles were demonstrated to possess antioxidant properties that supported cell survival and reduced the production of free radicals, as well as, were found to be non-toxic to neuronal (HT22) and macrophage (RAW164) cell lines (Schubert et al., 2006). In another study, xenon gas, a small molecule that can readily cross the BBB and has favorable neuroprotective properties, was loaded into liposomes and administered for up to 5 h after stroke onset with an optimal dosage range of 7–14 mg/kg and found to diminish infarct size in a rat model (Peng et al., 2013). Table 4 showing different types of nanocarriers used in acute ischemic stroke conditions.

**TABLE 4:** Nanocarriers for neuroprotective therapy in acute ischemic stroke.

Nanocarriers	Types of nanomaterials	Drugs/Agents	Targeting ligands	Outcomes	References
PNPs	PLGA	Tissue factor specific siRNA	EGFP-EGF1	Efficient RNA interference	Chen et al., 2013
	Glutathione coated PLGA-b-PEG	Thyroid hormones	Glutathione	Protection against ischemic damage	Mdzinarishvili et al., 2013
	PLGA	PEGylated epidermal growth factor, erythropoietin		Attenuation of inflammatory response and improved neurogenesis	Wang et al., 2013
	Chitosan	bFGF	Transferrin	Reduced infarct volume	Yemisci et al., 2015
	Gelatin	Osteopontin		Reduced infarct volume and extended therapeutic volume	Joachim et al., 2014
Liposomes	Cholesterol, PEG2000-PE	Minocycline		Reduced TNF-α induced MMP-9 release	Xing et al., 2012
	DPPC, cholesterol, PEG2000-PE, Egg-PC	Xenon		Reduced infarct size	Peng et al., 2013
	DSPC, DPPC, cholesterol	Tacrolimus		Reduced cerebral cell death, ameliorated motor function deficits	Fukuta et al., 2015
Metal and metal oxide		Platinum		ROS scavenging	Takamiya et al., 2012
		Cerium oxide		ROS scavenging	Estevez et al., 2011

*DPPC, 1,2-dipalmitoyl-sn-glycero-3-phosphocholine; DSPC, 1,2-distearoyl-sn-glycero-3-phosphocholine; Egg-PC, egg phosphocholine; MMP-9, matrix metalloproteinase-9; PEG2000-PE, 1,2-distearoyl-sn-glycero-3-phosphoethanolamine-N-[methoxy (polyethyleneglycol)-2000]; PLA, poly (lactic acid); PLGA, poly (D,L-lactide-co-glycolic acid); ROS, reactive oxygen species.*

## Nanotechnology Based Delivery of Neuroprotective Drugs

Free radicals, such as superoxide, hydroxyl, peroxynitrite, and peroxide, cause deleterious alterations in cells including DNA fragmentation, peroxidation of cell membrane lipids, mitochondrial energy disturbances and alteration in functionality of transporter proteins, they are considered as the main culprit having the key role in pathology of neurodegenerative, ischemic, and traumatic CNS disorders (Mahadik and Mukherjee, 1996; Dugan et al., 2001).

Neuroprotective compounds based on carbon-60 fullerene – the molecules having a 3-D pattern of evenly spaced carbon atoms, have been fabricated by the application of nanotechnological approach (Dugan et al., 2001). Fullerenols, hydroxyl functional group possessing fullerene derivatives, have been demonstrated to act as an antioxidant and free radical scavenger, due to which they are capable of reducing glutamate, N-methyl-d-aspartate (NMDA), Amino-3-hydroxyl-5-methyl-4-isoxazole-propionate (AMPA), and kainite induced excitotoxicity and apoptosis. The ability of these nano-structures to inhibit glutamate channels without affecting GABA (A) or taurine receptors as well as their capability to lower the glutamate-mediated intracellular calcium concentrations are the suggested mechanisms for fullerenol mediated neuroprotection (Jin et al., 2000; Dugan et al., 2001).

### Curcumin

Curcumin (diferuloylmethane), a biologically active and chief phenolic constituent of turmeric obtained from the rhizomes of *Curcuma longa Linn*, which in its crude form has been employed as a spice, dietary supplement, and as a component of various traditional medicines, has shown tremendous therapeutic efficacy in several disease (Chattopadhyay et al., 2008). Being a natural antioxidant, curcumin has been found to possess many pharmacological activities including anti-inflammatory, antimicrobial, anticancer, the neuroprotective effect in neurodegenerative disorders (Table 5), in both preclinical and clinical studies. Furthermore, curcumin has demonstrated hepatoprotective, nephroprotective, cardioprotective, neuroprotective, hypoglycemic, antirheumatic behavior, among which its neuroprotective action against various neurodegenerative disorders has attracted the attention of researchers. Despite the wide medicinal applications of curcumin, its clinical implication is hindered due to low solubility, physico-chemical instability, poor bioavailability, rapid metabolism, and poor pharmacokinetic (Chattopadhyay et al., 2008). However, these problems can be solved by developing efficient delivery system, and the nano-technological approach has provided a platform to handle all the hurdles for the efficient delivery and action of curcumin (Naksuriya et al., 2014).

**TABLE 5:** Evaluation of targeted curcumin nanoformulations for CNS therapeutics.

Nanoformulations	Target disease	Outcomes	References
Biodegradable PLGA-curcumin	Alzheimer’s Disease	Exhibit non-toxicity in human neuroblastoma SK-N-SH cells and protect from H_2_O_2_- induced rise in ROS. Able to prevent the induction of the redox-sensitive/transcription factor Nrf2 in the presence of H_2_O_2_, indicative approach to protect neurons against oxidative injury that is usually observed in AD	Doggui et al., 2012
PEG-liposomes with the anti-transferrin, lipid conjugate liposome, nanoliposomes, PEG–polylactic acid block copolymer	Alzheimer’s Disease	Aggregation inhibition of Aβ	Mourtas et al., 2014
tApoE3-conjugated and Curcumin loaded PBCA polymer nanoparticles	Alzheimer’s disease	Treatment of Aβ-induced cytotoxicity in AD	Mulik et al., 2010
Curcumin-gold nanoparticles	Alzheimer’s disease	Interacted with amyloid protein/peptide and simultaneously diminish amyloid fibrillation and dissolve amyloid fibrils by acting as artificial molecular chaperones	Palmal et al., 2014
Anti-amyloid antibody-Conjugated and curcumin/dexamethasone loaded gadolinium/magnetic nanoparticles	Alzheimer’s disease	Early diagnosis, effective targeting, and as a therapeutic agent(s) of cerebrovascular amyloid	Jaruszewski et al., 2014
PLGA-bPEG-triphenylphosphonium polymer (PLGA-b-PEG-TPP)-based curcumin nanoformulation	Huntington’s disease	Confirmed significant cytosolic and mitochondrial fractions in cells, indicating mitochondria-targeting chemotherapeutics	Marrache and Dhar, 2012
Solid-lipid-based curcumin nanoformulation	Huntington’s disease	Attenuated 3-nitropropionic-acid- induced Huntington’s disease in rats by increasing complex II activity, restoring the glutathione and superoxide dismutase	Sandhir et al., 2014
Poly (N-isopropyl acrylamide)–curcumin nanoformulation	Ischemic stroke	Improved neurobehavioral activity and reduced cytokine levels (tumor necrosis factor alpha (TNF-α) and IL-1β) and reduces oxidative stress	Ahmad et al., 2016
Solid lipid nanoparticles of curcumin	Ischemic stroke	Alleviated behavioral, oxidative, and nitrosative stress; acetylcholinesterase; and mitochondrial enzyme complex, and physiological parameters in cerebral ischemic reperfusion injury in rats	Kakkar et al., 2013

Different types of nano-carriers such as nanoparticles, micelles, nanocrystals, nano-emulsions, and nano-liposomes have fascinated the researchers in order to address problems related to poor bioavailability and pharmacokinetic of curcumin. In past few years, a lot of studies were carried out to find out the efficacy of various nanoformulations for better therapeutic activity. In a study, Joseph et al investigated the uptake and diffusion of curcumin loaded PLGA-PEG nanoparticles in neonatal rat brain. They observed neuroprotection against impaired blood–brain barrier in regions of injury. These nanoparticles diffuses effectively through the brain parenchyma (Joseph et al., 2018). In another study, Zhang et al. formulated curcumin-loaded polysorbate 80 modified cerasome (CPC) nanoparticles and delivered them to the MPTP-induced PD mice by ultrasound-targeted microbubble destruction of BBB. They reported better stability, longer circulation time, and higher permeation of nanoformulation of curcumin as compared to bulk curcumin. Furthermore, they observed improved behavior disorder along with dopamine depletion after treatment with CPC NPs (15 mg curcumin/kg) (Nisi Zhang et al., 2018). In *in-vitro* as well as *in-vivo* studies, PNPs encapsulated curcumin (NanoCurc^TM^) demonstrated better efficacy in protecting neurons from oxidative insults. NanoCurc^TM^ treatment protected human SK-N-SH cells from H_2_O_2_ mediated ROS insults. *In vivo*, intraperitoneal (IP) injection of NanoCurc^TM^ at a dose of 25 mg/kg twice daily in athymic mice demonstrated significant curcumin levels in the brain and resulted in decreased levels of H_2_O_2_, and caspases activities in brain along with increased glutathione concentrations (Ray et al., 2011). In a study, curcumin-encapsulated PLGA NPs (Cur-PLGA-NPs) demonstrated better effect on neural stem cell proliferation and neuronal differentiation *in vitro* as well as *in vivo*, as compared to bulk curcumin. Cur-PLGA-NPs significantly elevated expression of genes related to cell proliferation and neuronal differentiation in hippocampal region. They produced better effect on impaired learning and memory parameters in an Aβ induced rat model of AD. By *in silico* molecular docking studies it was found that these NPs induced neurogenesis through canonical Wnt/β-catenin pathway (Tiwari et al., 2013). Mathew et al synthesized a water soluble PLGA coated-curcumin NPs and coupled it with Tet-1 peptide, a peptide having high affinity to neurons. They found that curcumin encapsulated NPs successfully destroyed amyloid aggregates along with anti-oxidative property and non-cytotoxicity (Mathew et al., 2012).

### Growth Factors

Various growth factors among which, Nerve growth factors (NGFs) are most essential, are needed for the development and phenotype maintenance of neurons in the peripheral nervous system and for the functional integrity of neurons in the CNS. NGFs have great therapeutic potential for various CNS disorders (Table 6). Vascular endothelial growth factor (VEGF) was considered as a potentially useful therapeutic agent to attenuate ischemic brain injury, as it has been shown to participate in the process of post-ischemic brain repair via promoting neurogenesis and cerebral angiogenesis. Thus, effective neuroprotection and promotion of vascular regeneration in the ischemic brain have been achieved by treatment with VEGF loaded transferrin-modified liposomes (Tf-LPs). Zhao et al. (2010) achieved enhanced delivery of VEGF to the ischemic brain by encapsulated VEGF-encoding plasmids in Tf-LPs. It was found that the rats treated with Tf-LPs exhibited increased levels of VEGF mRNA and protein in the ischemic brain, compared with the rats treated with unmodified liposomes or saline.

**TABLE 6:** A brief summary of nanoformulations related to NGF.

Activity	Type of nanoparticle	Functional coating	Outcomes	References
Differentiation and survival	Gold nanorods	Coated with poly (4-styrenesulfonic acid) or SiO_2_	Increase the differentiation of NG108-15 cells	Paviolo et al., 2013
	Iron oxide	Conjugated to NGF	Stabilize NGF and enhance neuronal differentiation	Marcus et al., 2015
	Silver		Enhance the differentiation of SH-SY5Y cells	Alon et al., 2014
Directing Neuronal migration and growth	Iron oxide		Apply magnetic tensile forces to cause SH-SY5Y and primary Schwann cell cultures to migrate toward predefined directions	Riggio et al., 2012
	Iron oxide	Conjugated to NGF	Apply magnetic tensile forces to induce directed neurite sprout in PC12 cells	Riggio et al., 2014

### Edaravone

Edaravone (EDR), a well-known lipophilic drug, has shown great potential as a free radical scavenger for diseases including neurodegenerative disease, cardiovascular disease, and cancer. Recently, it has fascinated researchers as and has shown noteworthy pharmacological value against the incurable diseases like ALS and AD (Cruz, 2018). No oral formulation of the EDR is currently available in the market and liquid formulation for the purpose of intravenous infusion is the only commercially available formulation. Though, oral bioavailability of EDR is very less, yet, it has shown great potential for AD, cerebral aneurysm via oral administration in preclinical studies (Hudson et al., 2013). The EDR loaded lipid-based nanosystem (LNS) has been developed to facilitate its efficient oral delivery by augmenting the oral bioavailability. Solid-LNS showed elevated cellular uptake and better neuroprotective effect compared to EDR in SH-SY5Y695 cell line (Parikh et al., 2017).

## Obstacles to Clinical Translation

**FIGURE 6:**
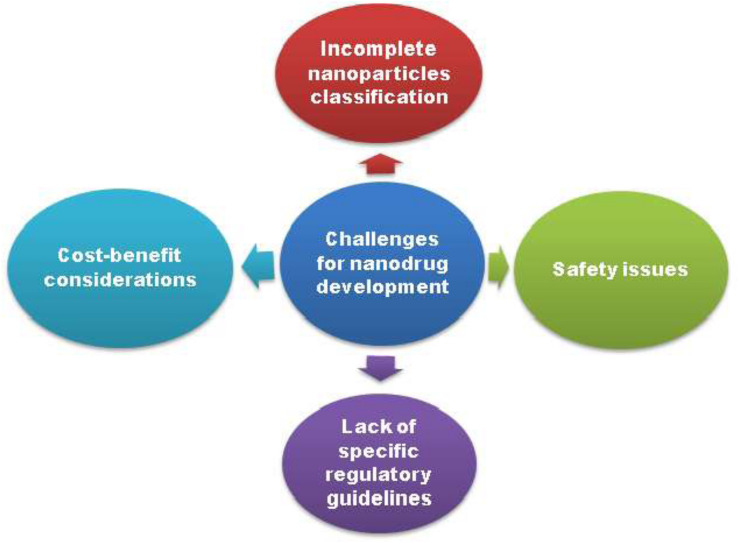
Various challenges for the nanotechnology-based drug delivery.

The emerging role of nanotechnology in therapeutics is not untouched by the challenges and does not always demonstrate promising results but also has been associated with several risks (Figure 6). Currently, nano-based approach is considered as a successful tool for delivery of drugs across BBB (Wong et al., 2012; Tajes et al., 2014; Havel et al., 2016). Although, a number of nanomedicine are in its infancy in the preclinical and clinical studies, and still sufficient data required to prove them as a better approach over conventional neurotherapeutics (Dinda and Pattnaik, 2013). The composition of nanocarrier is the key determinant of its properties, and may be responsible for oxidative stress, amino acid disturbance and BBB disruption that cause neurotoxicity in the brain (Sharma and Sharma, 2007). Although, nanoparticles functionalized with specific ligands provide successful drug targeting, but their extremely small size and the large surface area may cause problems like particle aggregation, interparticular friction, limited drug loading and high clearance rate sufficient to preclude their use in diagnosis and drug delivery.

The increased surface area results in an augmented chemical reactivity of the nanoparticles which then increase the production of ROS and ultimately leads to toxicity, such as neuroinflammation, excitotoxicity, DNA damage and allergic responses. Toxicity pattern of the nanocarriers depends on its state of aggregation, mechanical properties, mode of drug administration (Vega-Villa et al., 2008). Therefore, biocompatibility and biodegradability of nano-drugs are also critical to be fully understood. As nanomedicines have to interact with neurons to show their response, multidimensional interaction at neuronal level and restricted anatomical access increase the challenges in nano-based drug delivery (Jain, 2007).

The ultimate goal of nano-mediated CNS drug-delivery systems is to engineer the nanocarriers to be safe and to enable their long term use without any adverse side effects which made them successful in clinical translation from bench to bedside.

## Future Perspectives

Blood-brain barrier are the one of the pharmacologically active shield against CNS disorders. Brain-targeted drug delivery systems has been developed in last few recent years and gained large attention. The obstruction posed by BBB toward therapeutic drugs against diseased part of brain tissues, brain-targeted drug delivery systems seems to be the most promising strategy to address our natural defense system. A clear perceptive toward the functions of brain cells such as microglia, astrocytes, endothelial cells, and neural stem cells in neuro-disorders pathology is requisite in order to develop successful novel targeted-drug delivery systems. Even though, several nanoformulations have shown great efficacy in preclinical and clinical studies, their clinical translation from bench to bedside is not very successful due to inadequate information about their final fate in terms of toxicity, and other problems like aggregation and rapid clearance due to nano-size. In-depth and complete toxicological studies of brain targeting nanoformulations with their clear mechanism of action and pharmacokinetics with and without therapeutics should be essentially investigated. Besides this, more research has to be done in order to determine the fate of nanocarriers inside body, their systemic toxicity, biocompatibility, and RES elimination. The therapeutic potential of nanomedicine will rely on the rational approach and further designing of nanomaterials based on detailed and comprehensive knowledge of obtained from biological processes. Several basic concerns should be addressed in the future to achieve the successful clinical translation of nanoformulations:

(i) The nanomaterials should be biodegradable in nature and provide effective and safe brain-targeted drug delivery systems.
(ii) An eco-friendly green approach should be developed for preparation of nanoformulations.
(iii) The factors like shape, size, charge and moiety attached to nanomaterials should be well elucidated and evaluated, which is essential for crossing and developing brain-targeted drug delivery systems.
(iv) A non- invasive alternative method for nanocarrier drug delivery should be developed in order to avoid complications such as poor patient compliance associated with i.v., and other invasive routes. Newer drug-administration routes for nanocarrier-mediated CNS drug-delivery systems such as oral, transbuccal, mucosal/sublingual, or nasal, need to be explored.

The main issue for bench to bedside translation of nanoformulations is toxicity which has to be dealt carefully. The fate and mechanism of nanocarriers with respect to their bulk counterparts should be studied in detail before it comes to market. Therefore, in near future, fundamental research has to be carried out to deal with these issues if the successful efficient application of nanoformulations has to be achieved.

## Conclusion

Central nervous system diseases, such as AD, PD, stroke, brain tumors, and neuroinflammation have become distressful devastating to mankind due to the change in lifestyle and the rapid continued deterioration of the environment. BBB and BCSFB is the main physiological barrier which possess big bottleneck for the successful treatment of CNS disorders and brain tumors, complex anatomical structure, unique microenvironment, and their selectivity toward any foreign compound including drug is treated as a biggest challenge toward CNS drug delivery. High therapeutic drug concentration inside brain should be reached through effective and safe carrier, hence there is a need of developing good cargo, which carries drug to CNS in effective concentration without causing systemic side effects.

Nanomedicines have been implicated to address the problems related to treatment of neurological disorder and have a cutting edge over the conventional CNS therapy. Nanocarriers such as nanoparticles, liposomes, nanopharmaceuticals, nanotubes, nanoemulsions, nanosensors, dendrimers, and micelles, etc. are having high prospective in neuroprotection as theranostics. Engineered nanomaterials are designed by scientistist in order to increase its biocompatibility, blood circulation time and reduced their systemic toxicity, prevent the drug from degradation from physiological environment with sustained and controlled release along with the site-specific targeting. The current demand for the successful nano-based approaches focused on the regeneration and neuroprotection that will significantly benefit from continued nano based approach along with the advancement in neuronal cell biology their pathology and physiology. In models of AD, PD and stroke, different nanoscalic approaches have been identified to cure neurological disorders such as inhibition of Aβ oligomerization, reduce reactive oxygen species, and enhance functional neuronal networks. Nanocarriers have enabled targeted delivery of chemotherapeutics as well as antisense gene therapy, in malignant brain tumors, ensuing in remarkable inhibition of disease progression *in vitro* as well as *in vivo* study. Fortunately, a small number of these promising preclinical studies have been successfully translated to the clinic for effective patient care. Nanotechnology has been implicated in the development of nanoformulations of various neuroprotective drugs such as curcumin, nerve growth factors, and edaravone, etc. but still limited data are provided related to their adverse effects. Future perspective of use of nanotechnology in CNS drug delivery is very promising and it opens new avenues in the treatment of neurological disorders as it has the potential to fundamentally revolutionize the way we approach CNS-targeted therapeutics due to their ability to nanoengineered the drug/carriers to cross BBB, diffuse within the brain tissue, target specific cell, or signaling systems for delivering therapeutics.

## Author Contributions

AP wrote the initial first draft. SN conceptualized, proofread, edited, and wrote the first draft. SN and SF completed the final draft. NIPER-R communication no. is: NIPER-R/Communication/127.

## Conflict of Interest

The authors declare that the research was conducted in the absence of any commercial or financial relationships that could be construed as a potential conflict of interest.
